# Sex-related cardiac complications and their association on functional outcome in patients with aneurysmal subarachnoid hemorrhage: a retrospective study

**DOI:** 10.1007/s10143-025-03608-9

**Published:** 2025-05-29

**Authors:** Stephanie Koller, Massimo Barbagallo, Marco Luciani, Ignazio De Trizio, Francesca Casagrande, Federica Stretti, Vittorio Stumpo, Priska Eggenschwiler, Giuseppe Esposito, Emanuela Keller, Giovanna Brandi

**Affiliations:** 1 Institute for Intensive Care, University Hospital of Zurich and University of Zurich, Zurich, Switzerland; 2 Department of Cardiology, University Hospital of Zurich, Zurich, Switzerland; 3 Center for Experimental and Translational Cardiology, Schlieren, Zurich, Switzerland; 4 Department of Neurosurgery, Clinical Neuroscience Center, University Hospital Zurich and University of Zurich, Zurich, Switzerland

**Keywords:** Aneurysmal subarachnoid hemorrhage; cardiac complications; sex differences; functional outcome, Long-term outcome

## Abstract

Cardiac complications are common following aneurysmal subarachnoid hemorrhage (aSAH). Sex-related differences in the development of these complications and their association with short- and long-term outcome remain insufficiently understood. This retrospective study included all consecutive adult patients with aSAH who were treated at the neurocritical care unit (NCCU) at the University Hospital of Zurich between January 2016 and December 2022. Demographics, severity scores, laboratory and radiological findings as well as functional outcomes and mortality at different time points were analyzed. Furthermore, well-defined cardiac complications which arose during the NCCU stay were investigated. Data were dichotomized by sex, presence of cardiac complications and outcome (favorable vs. unfavorable). Based on the results of the univariate analysis and clinical relevance, multivariable logistic regression models were constructed to evaluate the simultaneous effects of multiple predictors on NCCU-mortality and functional outcome at 3 and 12 months. 387 patients were included (females n = 251 (64.9%)). Cardiac complications occurred frequently (n = 250 patients (64.6%)) in the acute phase and were more frequent in women (70.5% vs 53.7%, respectively, p = 0.001), in particular arrhythmic disorders (p < 0.001) and myocardial injuries (p = 0.045). With regard to long-term outcome, female sex and the development of cardiac complications were independently associated with an unfavorable outcome. Although female patients with aSAH may benefit from more intensive cardiac monitoring due to higher frequency of cardiac complications, our analysis also suggests that male patients who develop cardiac complications had a higher risk of an unfavorable long-term outcome than their female counterpart. Cardiac complications following aSAH are common in the acute phase. Women are more commonly affected, in particular from arrhythmias and myocardial injuries. Moreover, our data suggests that the acute development of cardiac complications and female sex may have an independent association with long-term unfavorable outcome. Male patients who develop cardiac complications have a higher risk of an unfavorable long-term outcome than their female counterpart.

**Supplementary Information:**

The online version contains supplementary material available at 10.1007/s10143-025-03608-9.

## Introduction

Cardiac complications, such as cardiac arrhythmias, electrocardiographic (ECG) changes, and neurogenic stunned myocardium, are common in patients with aneurysmal subarachnoid hemorrhage (aSAH) [1, 2]. They have been attributed to the activation of the sympathetic nervous system [3]. This induces catecholamines release, resulting in overstimulation of heart rate and cardiac contractility, increased systemic vascular resistance, and thus, stress on the heart. Additionally, the role of inflammation has been investigated, as additional contributor to cardiac dysfunction after aSAH[4]. Cardiac complications are associated with poor outcomes [5, 6], and increased risk of death [7] after aSAH.

Sex-related differences in patients with aSAH are well known with regard to epidemiology [8–11], anatomical aneurysm location [11, 12] and functional outcome [13]. The reasons for these differences are not completely understood. Sexual-hormonal differences, such as fluctuations in estrogen levels, childbirth, as well as anatomical differences in blood vessels, and social aspects seem to contribute [14, 15].

In contrast, sex-related differences in cardiac complications after aSAH have been scarcely investigated so far. In a previous work, we found that women with aSAH suffer more frequently than men from cardiac complications, in particular arrhythmia [16]. Data on sex-specific associations between cardiac complications and short- and long-term outcome following aSAH are missing.

This retrospective study was set out to analyze the sex-specific differences in cardiac complications following aSAH and their association with short- and long-term outcome. The identification of such differences might improve personalized management and treatment strategies.

## Material and methods

### Inclusion and exclusion criteria

This retrospective study included all consecutive patients with aSAH who were hospitalized at the Neurocritical Care Unit (NCCU) of the University Hospital of Zurich, between January 2016 and December 2022. Inclusion criteria were: 1) adults (≥ 18 years old); 2) with confirmed aSAH (either radiologically and/or through lumbar puncture); 3) signed informed consent by patient him-/herself, or, in case of incapacity of the patient due to the neurological impairment, by the next of kin. Exclusion criteria were: 1) patients with unruptured, traumatic, fusiform, dissecting, or mycotic aneurysms; 2) patients’ written or documented oral refusal to have their data analyzed for research projects.

This study was authorized by the Zurich Cantonal Ethics Committee (registration number: KEK 2022–00270) and was performed in accordance with the ethical standards laid down in the Declaration of Helsinki.

### Patient’s population and management

In case of hydrocephalus/ventriculomegaly, an external ventricular drain (EVD) was inserted. Early aneurysm securing through surgical or endovascular techniques was performed within 24 h from admission. Over a period of at least 14 days, patients were observed and treated at the NCCU or at the intermediate care unit due to the risk of vasospasm/delayed cerebral ischemia (DCI). Euvolemia and normal blood pressure were aimed to prevent DCI [10]. Body temperature was continuously monitored and in case of fever, surface- or intravascular temperature modulating systems were applied to reach normothermia. In case of symptomatic vasospasms (detected by computer tomography (CT) angiography with corresponding perfusion deficit), arterial hypertension was induced through administration of intravenous vasopressors. Patients who did not respond to the controlled hypertensive therapy were evaluated for selective intra-arterial vasodilator therapy with nimodipine or angioplasty. In case of a prolonged unconscious state, either due to the severity of the disease itself or due to the necessity for deep sedation to control intracranial pressure (ICP) during the vasospasm phase, an invasive multimodal neuromonitoring including cerebral microdialysis (CMA 70, CMA Mikrodialysis, Solna, Sweden), brain tissue oxygenation monitoring (LiCox system, Integra Neurosciences, Plainsboro, NJ), and continuous electroencephalography was inserted.

### Data collection

The medical records of all eligible patients were reviewed from the electronic clinical information system (KISIM-TM; Cistec,® Zurich, Switzerland) for demographics, cardiovascular risk factors, severity of bleeding, clinical and laboratory parameters, radiological findings on first head CT scan, intracranial complications, cardiac abnormalities during the NCCU stay, and short- and long-term outcomes. Demographic data included age, sex, localization of the ruptured aneurysm, pre-existing cardiac conditions, and comorbidities assessed with the Charlson Comorbidity Index (CCI) [17]. Current smoking and alcohol use disorders were also recorded as cardiovascular risk factors.

Severity of bleeding was assessed with the World Federation of Neurological Surgeons grades (WFNS)[18] and the modified Fisher scale[19]. For the analysis, WFNS was dichotomized into “high grade WFNS” (WFNS 4 and 5) and “low grade WFNS” (WFNS 1–3).

Pre-existing cardiovascular conditions included history of cardiovascular disease, arterial hypertension, heart failure, diabetes mellitus and valvular or arrhythmogenic heart disease, as found in medical reports.

Clinical and laboratory parameters analyzed included:

#### Laboratory findings

Creatine kinase (normal value < 170 U/l), myoglobin (normal value 25–58 mcg/l), high sensitive cardiac troponin (normal value < 14 ng/l), NT-proBNP (normal value < 249 ng/l). For the analysis, the highest values during the NCCU stay were considered.

#### Electrocardiogram (ECG) findings

ST segment relevant deviation, discordant T wave inversions, presence of Q wave, PQ/QRS/QTc time. The first ECG after hospital admission was considered for the analysis. ECG was interpreted by the NCCU doctors.

#### Echocardiographic (TTE) findings

(only the TTE performed by a specialist in cardiology during the NCCU stay were considered in the analysis): left ventricular ejection fraction (LV-EF), presence of wall motion abnormalities, diastolic disturbances, presence of morphological hints for pulmonary arterial hypertension. If available, previous echocardiographic evaluations were evaluated to determine whether the disorders were pre-existing.

Intracranial complications included rebleeding, radiological vasospasms, and DCI. DCI is defined as a clinical neurological deterioration (occurrence of a new focal deficit or the decrease of at least 2 points on the Glasgow Coma Scale (GCS) lasting for more than 1 h) and/or a new infarction revealed by CT imaging[20].

Cardiac complications during the NCCU stay were analyzed. These included acute myocardial injury[21], acute myocardial infarction[21], Takotsubo cardiomyopathy [22], arrhythmic disorders with new onset during the NCCU stay, and cardiac arrest with successful cardiopulmonary resuscitation. The definitions of the cardiac complications are available in the supplemental materials, Table S1.

The time of onset of cardiac complications was recorded and expressed in days after the initial bleeding.

Radiological findings on the first head CT scan included presence of subdural hematoma (SDH), intracerebral (ICH), or intraventricular hemorrhage (IVH), and hydrocephalus/ventriculomegaly.

Several outcomes were evaluated: NCCU length of stay (NCCU-LOS) expressed in days; Death during the NCCU stay; Frequency of immediate palliation (within 24 h from NCCU admission); Frequency of redirection of care to palliation during the NCCU stay. Functional outcome was evaluated using the Glasgow Outcome Scale Extended (GOSE)[23] at hospital discharge, 3- and 12 months after the initial bleeding and was assessed by a NCCU doctor based on the follow up visits and/or from the discharge letters from the rehabilitation clinics. GOSE was dichotomized as *favorable* (GOSE 5 to 8) and *unfavorable* (GOSE 1 to 4) [16, 20, 24].

### Statistical analysis

Statistical analysis was performed using R, version 4.3.3. Data were dichotomized by sex (male vs. female), cardiac complications (presence or absence of cardiac complications during the NCCU-stay), and outcome (favorable vs. unfavorable). Descriptive statistics were reported as counts/percentages, mean ± standard deviation (SD), or as median including the quartiles 1 and 3 (Q1-Q3), as appropriate. The level of significance was defined as the probability for a type I error of less than 5%, corresponding to a p-value of < 0.05. Categorical variables were compared using the Fisher’s exact test. For continuous variables, the Student’s t-test was applied, if they were normally distributed, while Kruskal–Wallis test in the case of non-normally distributed variables. To visualize survival and occurrence of cardiac complications, Kaplan–Meier survival curves were created.

Outcomes of interest were binarized as follows: death during ICU care, unfavorable GOSE score at 3 months, unfavorable GOSE score at 12 months and high/low grade WFNS, in order to assess the relevance of possible prognostic factors at different time points during patients’ follow-up. Patients with missing outcome data were excluded and patients who died at the ICU were excluded from the analysis at later time points (i.e. 3 and 12 months). Missing values in predictor variables were handled using multiple imputation by chained equations (MICE) with predictive mean matching. In addition to univariate analysis previously performed, univariable logistic regression was additionally performed to further assess the association of individual predictors with each outcome. Univariate logistic regression models were constructed for each predictor-outcome pair, and odds ratios (ORs) with 95% confidence intervals (CIs) were calculated. Initially predictors of interest were selected based on clinical knowledge and statistical significance at univariate statistics. Multivariable logistic regression models were constructed to evaluate the simultaneous effects of multiple predictors on each outcome. An interaction term, specifically “cardiac complications * sex”, was included to assess effect modification of sex on cardiac complications in affecting the three selected outcomes. Model diagnostics were performed to ensure robustness as follows: Variance Inflation Factor (VIF) to detect multicollinearity among predictors; Hosmer–Lemeshow Test, to assess goodness-of-fit; ROC Curve analysis, to evaluate model discrimination, with the area under the curve (AUC) reported. Backward stepwise selection was employed to refine multivariable models. Predictors were iteratively removed based on the Akaike Information Criterion (AIC) to identify the most parsimonious model. Final multivariable logistic regression models were summarized, and significant predictors were reported with ORs, 95% CIs, and p-values. For visualization, ROC curves and forest plots of the ORs for each variable included in the final model were created.

## Results

Overall, 387 patients fulfilled the inclusion criteria, 64.9% of them were women (n = 251). Demographic data are summarized in Table 1. Women were older than men (59 [50, 71] vs 54 [47, 62] years, (p = 0.001), respectively). Furthermore, more men were current smokers (p = 0.001) and presented alcohol use disorders (p = 0.02) at the time of the aSAH. Men and women did not differ for frequency of comorbidities, as expressed by the CCI, and for frequency of pre-existing cardiac diseases, as shown in Table 1.

**Table 1:** Demographics, pre-existing cardiac conditions, cardiovascular risk factors

	Overall	Male	Female	p value
Sex n (%)	387 (100.0)	136 (35.1)	251 (64.9)	
Age, years (median [Q1-Q3])	57 [49—68]	54 [47—62]	59 [50—71]	0.001
CCI total (median [Q1-Q3])	0.00 [0.00—2.00]	0.00 [0.00—2.00]	0.00 [0.00–1.00]	0.991
History of any heart disease (%)	184 (47.8)	66 (48.9)	118 (47.2)	0.834
Coronary heart disease (%)	12 (3.1)	6 (4.4)	6 (2.4)	0.427
Heart failure (%)	1 (0.3)	0 (0.0)	1 (0.4)	1
Arterial hypertension (%)	174 (45.2)	62 (45.9)	112 (44.8)	0.917
Valvular disease (%)	16 (4.2)	5 (3.7)	11 (4.4)	0.953
Rhythmogenic heart disease (%)	10 (2.6)	4 (3.0)	6 (2.4)	0.994
Diabetes mellitus (%)	19 (7.6)	9 (12.3)	10 (5.6)	0.121
Current smoking (%)	178 (53.8)	75 (63.6)	103 (48.4)	0.011
Alcohol use disorder (%)	13 (3.4)	9 (6.6)	4 (1.6)	0.02

Table 1 Demographics, pre-existing cardiac conditions, cardiovascular risk factors. Comparisons among male and female patients with aneurysmal subarachnoid hemorrhage were performed. Abbreviations: Q1-Q3: quartiles 1 and 3; CCI: Charlson Comorbidity Index

In addition, no differences were found in treatment modalities for aneurysm securing, as well as frequency of intracranial complications, as shown in Table 2. Sex-related differences in aneurysm localization are presented in Table 2. More men than women suffered from subdural hematoma (p = 0.037), otherwise no other differences were found in radiological findings on first head-CT scan, as presented in Table 2

**Table 2:** Aneurysm localization, severity scores, and intracranial complications

	Overall (N = 387)	Male (n = 136)	Female (n = 251)	p value
*Aneurysm localization*				0.03
Internal carotid artery	31 (8.0)	11 (8.1)	20 (8.0)	
Middle cerebral artery	96 (24.8)	31 (22.8)	65 (25.9)	
Anterior communicating artery	116 (30.0)	57 (41.9)	59 (23.5)	
Anterior cerebral artery	6 (1.6)	1 (0.7)	5 (2.0)	
Vertebral artery	12 (3.1)	4 (2.9)	8 (3.2)	
Posterior cerebral artery	4 (1.0)	1 (0.7)	3 (1.2)	
Posterior inferior cerebellar artery	20 (5.2)	5 (3.7)	15 (6.0)	
Anterior inferior cerebellar artery	1 (0.3)	1 (0.7)	0 (0.0)	
Posterior communicating artery	56 (14.5)	11 (8.1)	45 17.9)	
Pericallosal artery	12 (3.1)	4 (2.9)	8 (3.2)	
Basilar artery	24 (6.2)	8 (5.9)	16 (6.4)	
Superior cerebellar artery	5 (1.3)	1 (0.7)	4 (1.6)	
Anterior choroidal artery	3 (0.8)	0 (0.0)	3 (1.2)	
Unknown	1 (0.3)	1 (0.7)	0 (0.0)	
Anterior circulation (%)	320 (82.8)	115 (84.5)	205 (81.7)	
Posterior circulation (%)	66 (17.1)	20 (14.6)	46 (18.4)	
*Bleeding severity scores*				
WFNS (%)				0.52
Score 1	126 (32.6)	42 (30.9)	84 (33.5)	
Score 2	80 (20.7)	28 (20.6)	52 (20.7)	
Score 3	21 (5.4)	4 (2.9)	17 (6.8)	
Score 4	75 (19.4)	30 (22.1)	45 (17.9)	
Score 5	84 (21.7)	32 (23.5)	52 (20.7)	
WFNS dichotomized (%)				0.236
Low grade WFNS	227 (58.8)	74 (54.4)	153 (61.2)	
High grade WFNS	160 (41.2)	63 (45.6)	98 (38.8)	
Hunt and Hess (%)				0.881
1	83 (21.5)	29 (21.3)	54 (21.6)	
2	96 (24.9)	32 (23.5)	64 (25.6)	
3	68 (17.6)	22 (16.2)	46 (18.4)	
4	67 (17.4)	27 (19.9)	40 (16.0)	
5	71 (18.4)	26 (19.1)	45 (18.0)	
N/A	1 (0.3)	0 (0.0)	1 (0.4)	
mFisher (%)				0.536
1	19 (4.9)	6 (4.4)	13 (5.2)	
2	22 (5.7)	7 (5.1)	15 (6)	
3	97 (25.1)	40 (29.4)	57 (22.7)	
4	247 (63.8)	83 (61)	164 (65.3)	
N/A	2 (0.5)	0 (0.0)	2 (0.8)	
*Radiological findings*				
Intracerebral hemorrhage (%)	104 (26.9)	40 (29.4)	64 (25.5)	0.478
Hydrocephalus/Ventriculomegaly (%)	210 (54.3)	72 (52.9)	138 (55.0)	0.781
Intraventricular hemorrhage (%)	271 (70.2)	90 (66.2)	181 (72.1)	1.000
Subdural hematoma (%)	34 (8.8)	18 (13.2)	16 (6.4)	0.037
*Treatment modalities*				0.608
Conservative	14 (3.6)	6 (4.4)	8 (3.2)	
Endovascular	194 (50.1)	64 (47.1)	130 (51.8)	
Surgical	179 (46.3)	66 (48.5)	113 (45.0)	
*Intracranial complications:*				
Re-rupture (%)	2 (0.5)	1 (0.7)	1 (0.4)	1.000
Radiological vasospasm (%)	207 (60.0)	74 (59.2)	133 (60.5)	0.909
Vasospasm with perfusion deficits (%)	105 (27.1)	28 (20.6)	77 (30.7)	0.044
Delayed cerebral ischemia (%)	98 (28.4)	39 (31.2)	59 (26.8)	0.457

Table 2 Comparisons among females and males patients with aneurysmal subarachnoid hemorrhage on aneurysm localization, bleeding severity scores at hospital admission, radiological findings on first brain CT scan, treatment modalities and complications. Abbreviations: mFisher: Modified Fisher scale, WFNS: World Federation of Neurosurgical Societies grade, NA: not available

Overall, 250 patients (65%) developed cardiac complications during the NCCU stay, mostly within the first 72 h following aSAH (in supplemental materials, Fig. 1). Women were more likely than men to develop them (70.5 vs 53.7%, respectively, p = 0.001), as shown in Table 3. In particular, women were more likely than men to develop arrhythmic disorders (p < 0.001) and myocardial injuries as revealed by higher cardiac troponins (p = 0.045),  as shown in Table 3

**Table 3:** Cardiac abnormalities during the NCCU stay

	Overall (n = 387)	Male (n = 136)	Female (n = 251)	p value
*Cardiac Complications any kind (%)*	250 (65)	73 (54)	177 (70)	0.001
*Arrhythmic disorders any kind (%)*	182 (47)	46 (34)	136 (54)	< 0.001
Atrial fibrillation (%)	25 (6)	7 (5)	18 (7)	0.578
*ECG changes*				
ECG abnormalities (%)	159 (46)	63 (52)	96 (43)	0.146
ST segment abnormality (%)	86 (25)	41 (36)	45 (20)	0.008
T wave abnormality (%)	56 (16)	16 (13)	40 (18)	0.321
PQ time in ms (mean (SD))	157(28)	161 (31)	155 (26)	0.065
QRS time in ms (mean (SD))	87 (16)	93(18)	84(13)	< 0.001
QTc time in ms (mean (SD))	450 (38)	437 (36)	458(36)	< 0.001
AV blockage (%)	11 (2.9)	3 (2.2)	8 (3.3)	0.785
*Echocardiography findings*				
Availability of Echocardiography (%)	141 (36)	48 (35)	93 (37)	0.816
LV-EF absolute in % (mean (SD))	59 (10)	59 (8)	59 (11)	0.908
LV-EF below 50% (%)	22 (15.6)	6 (12.5)	16 (17.2)	0.628
LV wall abnormality (%)	36 (25.7)	12 (25.0)	24 (26.1)	1
*Blood tests*				
CK peak (median [Q1-Q3])	282 [153—516]	382 [223—688.]	230.5 [124- 429]	< 0.001
Troponin T peak (median [Q1-Q3])	22 [9—97]	15.5 [8—57]	27 [10—107]	0.045
Myoglobin peak (median [Q1-Q3])	71 [35—180]	106 [42—264]	65 [32—1665]	0.006
NT-proBNP peak (median [Q1-Q3])	743 [316- 2077]	573 [218—1293]	854 [391—2311]	0.004
Acute myocardial infarction (%)	22 (6)	10 (7)	12 (5)	0.398
Acute myocardial injury (%)	172 (44)	56 (41)	116 (46)	0.541
Takotsubo cardiomyopathy (%)	21 (5)	3 (2)	18 (7)	0.068

Table 3 Cardiac complications that arose during the NCCU-stay. Comparisons among males and females with aneurysmal subarachnoid hemorrhage

Abbreviation: AV: Atrioventricular, CK: Creatinine kinase; ECG: Electrocardiogram, LV: Left ventricular, EF: ejection fraction, NT-proBNP: N-terminal pro–B-type natriuretic peptide.SD: Standard Deviation

Regarding outcomes, men and women had comparable NCCU-LOS (p = 0.854), as shown in Table 4. During the NCCU-stay, 13% of the patients died irrespectively of sex (p = 0.582). Immediate palliation occurred similarly in men and women (p = 0.815), as well as the withdrawal of life sustaining treatments during the NCCU-stay (p = 0.376). Functional outcomes, as assessed with the GOSE and after dichotomization in favorable and unfavorable, did not differ among men and women over time, as shown in Table 4. The follow-up data for GOSE at 3 months (338 patients) and at 12 months (302 patients) maintained an excellent consistency of relative female/male ratio (64.2% female versus 35.8% males at 3 months and 63.2% female versus 36.8% male at 12 months).

**Table 4:** Survival and functional outcomes in patients with aneurysmal subarachnoid hemorrhage

	Overall (n = 387)	Male (n = 136)	Female (n = 251)	p value
Days in NCCU (median [Q1-Q3])	15.00 [11.00—22.00]	14.50 [12.00—21.00]	15.00 [11.00—23.00]	0.854
Died during NCCU stay (%)	49 (12.7)	15 (11.0)	34 (13.5)	0.582
Immediate palliation (%)	11 (2.8)	3 (2.2)	8 (3.2)	0.815
Wiithdrawal of life sustaining treatments during NCCU-stay (%)	43 (11.1)	12 (8.8)	31 (12.4)	0.376
GOSE at 3 months (median [Q1-Q3])	5.00 [3.00—6.00]	5.00 [3.00—6.00]	4.00 [3.00—7.00]	0.422
GOSE at 12 months (median [Q1-Q3])	5.00 [3.00—7.00]	6.00 [3.00—7.00]	5.00 [3.00—7.00]	0.348
Favorable outcome at 3 months, n (%)	201 (51.9)	80 (58.8)	121 (48.2)	0.059
Favorable outcome at 12 months, n (%)	211 (60.6)	80 (64.0)	131 (58.7)	0.396

Table 4 Survival and functional outcomes in patients with aneurysmal subarachnoid hemorrhage. Comparisons among male and female patients with aneurysmal subarachnoid hemorrhage on frequency of death, redirection of care, and functional outcome, as assessed with the Glasgow Outcome Scale Extended (GOSE) at different time points. Favorable outcome was defined as GOSE 5 to 8. Abbreviations: GOSE: Glasgow Outcome Scale Extended, NCCU: Neurocritical care unit, Q1-Q3: quartiles 1 and 3

**Fig. 1:**
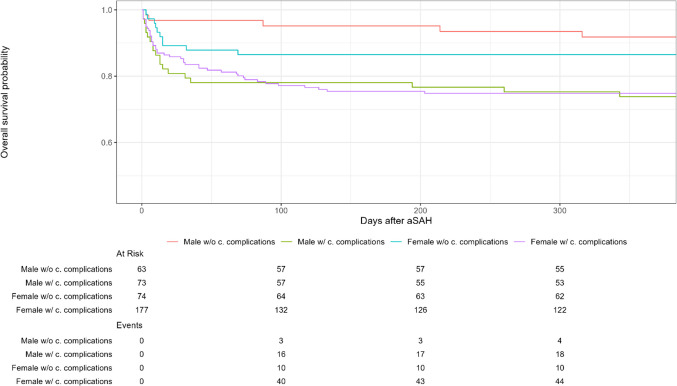
Survival curves of male and female patients with and without cardiac complications. Survival curves for male and female NCCU survivors who developed cardiac complications during the NCCU- stay (green and purple curves, respectively) and for male and female NCCU survivors who did not (red and turquoise curves, respectively). NCCU: neurocritical care unit

Patients who developed cardiac complications during the NCCU-stay had a worse long–term outcome than patients who did not for both sexes (Fig. 1).

Comparing patients with (n = 250) and without (n = 137) cardiac complications, patients with cardiac complications were older (61 [52, 71] vs 52 [45, 59] years, (p < 0.001), respectively) and more likely to have pre-existing cardiac disease and comorbidities, based on the CCI (1 [0,2] vs 0[0,1] respectively, p < 0.001). Demographic data are summarized in supplemental materials Table 2.

No differences were found in frequency of current smokers and alcohol use disorders at the time of the aSAH (supplemental materials Table 2).

In addition, no differences were found in treatment modalities for aneurysm securing, but patients with cardiac complications were more likely to receive an EVD (p < 0.001). Considering the intracranial complications, patients with cardiac complications were more likely to develop vasospasms (p = 0.034), and vasospasms with perfusion deficits (p = 0.038), as shown in supplemental materials Table 3. More patients with cardiac complications than patients without suffered from ICH (p = 0.046), hydrocephalus (p < 0.001) and intraventricular hemorrhage (p < 0.001), as presented in supplemental materials Table 3.

Regarding outcomes, patients with cardiac complications had longer NCCU-LOS than patients without (17 [Q1-Q3 12—27] vs 13 [Q1-Q311—16] days, (p < 0.001), respectively), as shown in supplemental materials Table 4. During the NCCU-stay, 16% of the patients with cardiac complications died compared to 7% of patients without (p = 0.029). Immediate palliation occurred similarly in both groups (p = 0.373), while the decision of redirection of care to palliation during the NCCU-stay was more frequent for patients with cardiac complications (p = 0.009). Functional outcomes, as assessed with the GOSE and after dichotomization in favorable and unfavorable, was worse in patients with cardiac complications, as shown in supplemental materials Table 4.

Considering the analysis by sex and by occurrence of cardiac complications, both male and female patients with cardiac complications were older and with more severe bleeding than male and female patients without**.**

**Fig. 2:**
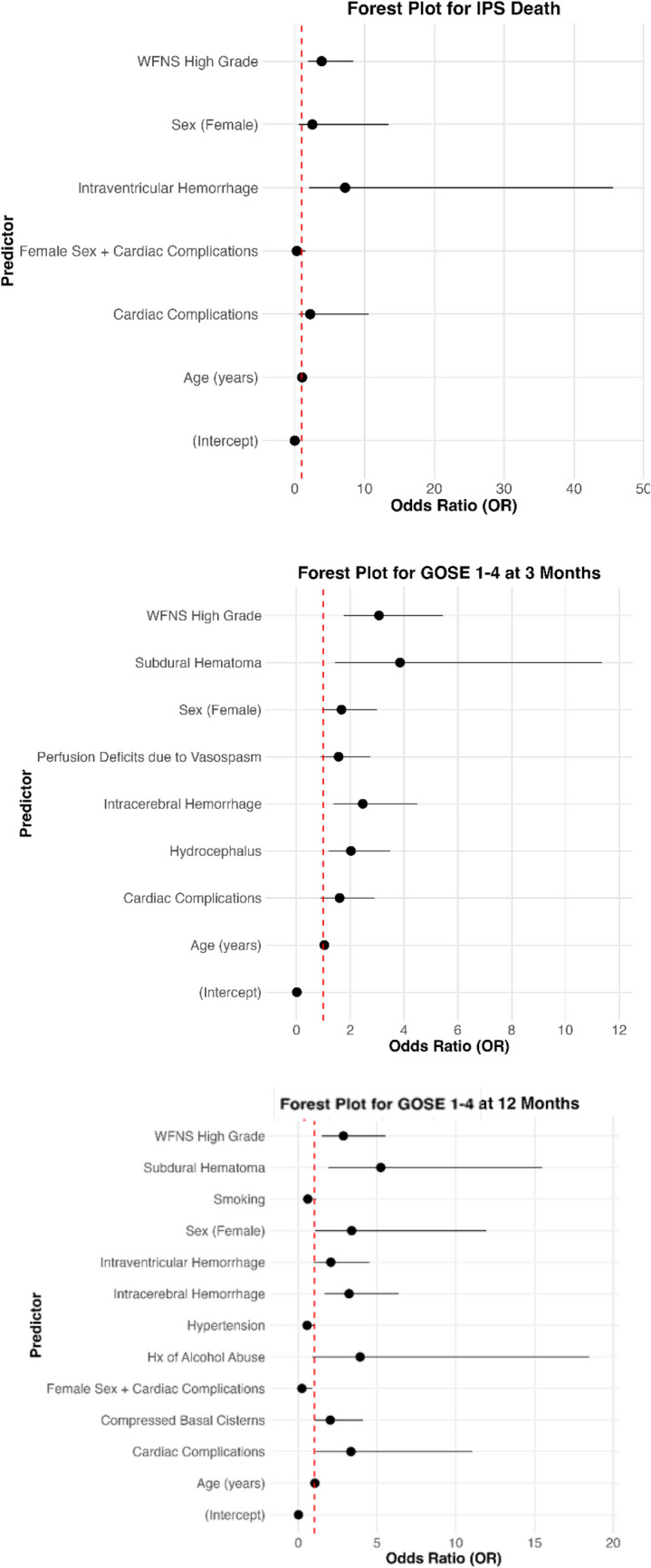
Forest plots for NCCU-mortality (above), unfavorable outcome at 3 (in the middle) and 12 months

In the multivariate analysis, NCCU death was independently associated with older age, high grade WFNS, and presence of intraventricular hemorrhage in the first head CT scan (Table 5 and Fig. 2). At 3 months post aSAH, unfavorable outcome was independently associated with increasing age, high grade WFNS, presence of ICH, SDH and hydrocephalus in the first head CT scan (Table 5 and Fig. 2). Considering the long-term outcome, unfavorable outcome at 12 months following aSAH was associated with female sex, increasing age, high grade WFNS; presence of ICH, SDH, compressed basal cisterns in the first head CT scan, and development of cardiac complications during the NCCU-stay. Interestingly, being female and at the same time having developed a cardiac complication during the NCCU-stay was associated with a decreased OR of negative GOSE outcome (1–4) at 12 months i.e. protective effect, with respect to male patients who developed cardiac complications (Table 5 and Fig. 2). ROC analysis revealed high discrimination of the final multivariable models with AUC of 0.806 (95%CI 0.747–0.864) for NCCU death, AUC of 0.807 (95% CI 0.761–0.854) for GOSE 1–4 at 3 months and AUC of 0.825 (95% CI 0.776–0.874) for GOSE 1–4 at 12 months (in supplemental materials, Fig. S2).

**Table 5:** Multivariable models

ICU death							
	Univariate Analysis				Multivariate Analysis		
Variable	Estimate	OR (95%CI)	P value	Estimate		OR (95%CI)	P value
Sex	0.23	1.26 (0.67–2.48)	0.47	0.92		2.52 (0.57–13.43)	0.23
Age	0.05	1.05 (1.03–1.08)	< 0.001	0.05		1.05 (1.02–1.08)	< 0.001
high grade WFNS	1.58	4.84 (2.53–9.80)	< 0.001	1.34		3.85 (1.88–8.35)	< 0.001
Intraventricular hemorrage	2.46	11.80 (3.56–73.06)	< 0.001	1.98		7.21 (2.07–45.6)	< 0.01
Cardiac complications	0.85	2.34 (1.17–5.12)	< 0.05	0.79		2.2 (0.61–10.61)	0.26
Interaction factor	-	-	-	−1.23		0.29 (0.04–1.54)	0.15
GOSE 1–4 at 3 months							
Sex	0.45	1.58 (0.99–2.52)	0.053	0.51		1.67 (0.95–2.99)	0.07
Age	0.04	1.04 (1.02–1.06)	< 0.001	0.04		1.04 (1.01–1.06)	< 0.001
WFNS > 3	1.62	5.04 (3.14–8.18)	< 0.001	1.12		3.07 (1.75–5.44)	< 0.001
Intracerebral hemorrhage	1.32	3.75 (2.26–6.35)	< 0.001	0.90		2.46 (1.37–11.36)	< 0.01
Subdural hematoma	1.54	4.67 (2.00–12.22)	< 0.001	1.35		3.85 (1.44–11.36)	< 0.01
Hydrocephalus	1.13	3.10 (1.98–4.91)	< 0.001	0.71		2.02 (1.18–3.48)	< 0.01
Vasospasms with perfusion deficits	0.67	1.95 (1.20–3.17)	< 0.01	0.45		1.57 (0.90–2.74)	0.11
Cardiac complications	1.22	3.39 (2.09–5.60)	< 0.001	0.47		1.60 (0.89–2.91)	0.12
GOSE 1–4 at 12 months							
Sex	0.14	1.15 (0.69–1.95)	0.58	1.22		3.38 (1.05–11.95)	< 0.05
Age	0.04	1.04 (1.02–1.07)	< 0.001	0.05		1.04 (1.02–1.07)	< 0.001
WFNS > 3	1.59	4.93 (2.93–8.43)	< 0.001	1.05		2.86 (1.49–5.54)	< 0.001
Intracerebral hemorrhage	1.38	3.96 (2.30–6.87)	< 0.001	1.16		3.22 (1.65–6.37)	< 0.001
Subdural hematoma	1.78	5.94 (2.53–15.10)	< 0.001	1.65		5.22 (1.90–15.48)	< 0.01
Compressed basal cisterns	0.75	2.11 (1.24–3.67)	< 0.01	0.70		2.02 (1.03–4.09)	< 0.05
Intraventricular hemorrhage	1.05	2.86 (1.59–5–40)	< 0.001	0.72		2.06 (0.98–4.53)	0.06
History of alcohol abuse	1.06	2.89 (0.93–9.24)	0.06	1.36		3.92 (0.88–18.47)	0.08
Cardiac complications	0.95	2.60 (1.52–4.60)	< 0.001	1.20		3.34 (1.10–11.04)	< 0.05
Hypertension	0.18	1.20 (0.73–1.98)	0.46	−0.59		0.55 (0.28–1.06)	0.08
Smoking	−0.25	0.78 (0.47–1.27)	0.31	−0.52		0.59 (0.30–1.13)	0.11
Interaction factor	-	-	-	−1.51		0.22 (0.05–0.88)	< 0.05

Table 5 Univariable and multivariable logistic regression models with NCCU-death, unfavorable outcome at 3 and 12 months as dependent variables. OR odds ratio

## Discussion

In the present study, we investigated sex-related differences in cardiac complications after aSAH and their possible associations with short- and long-term mortality. As main findings, we found that cardiac complications occur more frequently in women than in men, and above all in the first days after aSAH, despite a relatively low prevalence of prior cardiac history. In particular, women more frequently developed arrhythmic disorders and myocardial injury. Moreover, our data suggest that the acute development of cardiac complications and female sex may have an association with long-term unfavorable outcome.

Interactions between heart and brain have already been investigated in the past. Increasing evidence suggests that acute cerebrovascular events not only coincide with cardiac pathology due to common risk factors, but instead they may provoke cardiac dysfunctions [25–28]. Suggested physio-pathological pathways are an impairment of the autonomic nervous system leading to cardiac damage, as well as activation of the systemic inflammatory response [4].

Previous studies have already focused on arrhythmic disorders and elevations in cardiac enzymes following aSAH [29–32]. As in our study, they found that electrocardiography changes, such as QT prolongation, ST segment changes, and T wave inversions occur in most patients with aSAH. Furthermore, similarly to our results, they reported that patients with aSAH are at high risk for arrhythmias, ranging from sinus bradycardia to atrial fibrillation, atrial flutter and other supraventricular tachycardia. Additionally, it has been shown that elevated cardiac troponins are seen in 20–46% patients shortly after aSAH. Our results not only confirm these previous findings, but also suggest that female patients with aSAH are more likely to develop these abnormalities than men. This new finding is worthy of further investigation. From a practical point of view, it suggests that female patients with aSAH could benefit more than men from more intensive cardiac monitoring. The reason why women have more cardiac complications than men following aSAH could be due to the sex-specific activation of the autonomic nervous system [33–35], which could predispose women to an increased risk. Sex-related differences in management could also play a role, but in this context they seem to be less relevant, as previously reported [20]. Most cardiac complications in the study population occurred already in the first 24 h after aSAH, suggesting they were more likely due to the acute neurological event rather than differences in management.

The generated multivariable logistic regression models displayed excellent discriminative performance highlighting the relevance of included variables in prognosis prediction. In particular, in the multivariable models, NCCU-death was associated with well-known predictors for mortality as increasing age [36, 37], elevated WFNS grade [18], and presence of intraventricular bleeding[38]. At this time point being female and the development of cardiac complications during the NCCU stay were not associated with death. These results suggest that the severity of initial bleeding appears to be more relevant than sex and cardiac complications for NCCU death. With regard to long-term outcome, on the other hand, in addition to the already established well-known predictors, female sex and the development of cardiac complications are predisposing factors for an unfavorable outcome. Surprisingly, being female and at the same time having had cardiac complications seems to be protective against an unfavorable outcome with respect to male patients who develop cardiac complications. One possible explanation could be that the female population examined is relatively young and includes women with different hormonal states (premenopausal, menopausal and post-menopausal). The protective role of estrogens—lasting up to 5–20 years after the onset of menopause [34, 35, 39] – could possibly outweigh the possible negative effect of cardiac complications. This speculation unfortunately cannot be verified by analyzing patients according to age and sex because of the small sample size. Another possible explanation is that women after the aSAH may also have been exposed differently than men in terms of protections and vulnerability. But again to test this hypothesis we are limited by the sample size and the lack of data on e.g. cardiovascular risk. 

This study has several strengths. Firstly, we used a database of consecutive patients with aSAH during a 7-year period with several collected parameters and long-term follow-ups. Secondly, although the interactions between heart and brain have already been investigated, very little data exist on sex-specific interactions between the two organs. Thus, our findings are new. Thirdly, data on patients’ baseline characteristics, pre-existing medical conditions, radiological findings, as well as intracranial complications and outcomes are complete, with only few missing data.

Some limitations should be mentioned. Firstly, this is a single center experience, thus limiting the generalizability of our findings. Secondly, due to the retrospective nature of the study, the cardiac complications were extracted from the medical reports. A form of selection bias cannot be ruled out. Thirdly, we are not able to identify which specific arrhythmias were more frequent in female patients, also due to the small sample size. Fourthly, less than half of patients received an echocardiography performed by the cardiologists during their NCCU-stay, thus possibly preventing the identification of sex-specific alterations in cardiac function. Furthermore, we relied on the cardiologist's judgement for the TTE evaluation, this leaves room for a certain operator's dependency, as for example in the distinction between LV wall abnormality and an atypical form of Takotsubo cardiomyopathy. Fifthly, data on cardiovascular profile are few. Lastly, due to the small sample size, we cannot conduct a detailed analysis to test for long-term interactions between female sex, age, cardiac complications and outcome.

In conclusion: Cardiac complications following aSAH are frequent, occur mostly in the very acute phase and they seem to be negatively associated with long-term outcome. Women are more likely than men to develop cardiac complications, in particular arrhythmic disorders and myocardial injury. This might be due to the sex-specific activation of the autonomic nervous system. Although female patients with aSAH may benefit from more intensive cardiac monitoring due to higher frequency of cardiac complications, our analysis also suggests that male patients who develop cardiac complications have a higher risk of an unfavorable long-term outcome than their female counterpart. Further research with larger sample size and appropriate age distribution is needed to clarify which and if any factors (e.g. hormonal) play a role in determining a protective effect in female patients despite the higher incidence of cardiac complications and overall worse prognosis.

## Supplementary Information

Below is the link to the electronic supplementary material.

Supplementary file1 (DOCX 733 KB)

## Data Availability

No datasets were generated or analysed during the current study.

## Abbreviations

aSAH: Aneurysmal subarachnoid hemorrhage
AIC: Akaike Information Criterion
AUC: Area Under the Curve
CCI: Charlson Comorbidity Index
CI: Confidence Interval
CT: Computer Tomography
DCI: Delayed Cerebral Ischemia
ECG: Electrocardiogram
EVD: External ventricular drain
GCS: Glasgow Coma Scale
GOSE: Glasgow Outcome Scale Extended
ICH: Intracerebral Hemorrhage
ICP: Intracranial Pressure
ICU: Intensive Care Unit
IVH: Intraventricular Hemorrhage
NCCU: Neurocritical Care Unit
NCCU-LOS: Neurocritical Care Unit -length of stay
OR: Odds Ratio
Q1-Q3: Quartiles 1 and 3
SD: Standard deviation
SDH: Subdural Hematoma
WFNS: World Federation of Neurological Surgeons Scale
LV-EF: Left Ventricle Ejection Fraction
MICE: Multiple Imputation by Chained Equations
