# Supplementary material for: Sex-related cardiac complications and their association on functional outcome in patients with aneurysmal subarachnoid hemorrhage: a retrospective study
Source: Neurosurg Rev. 2025 May 29;48(1):461. doi: 10.1007/s10143-025-03608-9 (PMC12122586; doi:10.1007/s10143-025-03608-9)
Supplement: Supplementary file 1 — Supplementary file1 (DOCX 733 KB) [file 10143_2025_3608_MOESM1_ESM.docx]

**Supplemental materials**

**Table 1.**

**Definitions of cardiac complications analyzed:**

| **Acute myocardial injury**: Elevation of cardiac troponin with at least one value above the 99th upper reference limit, with a newly detected rising and/or falling pattern [21] |
| --- |
| **Acute myocardial infarction**: Acute myocardial injury and at least one of the following: clinical symptoms of myocardial ischaemia, new ischaemic ECG changes, development of pathological Q waves, new regional wall motion abnormality in a pattern consistent with an ischaemic aetiology, identification of a coronary thrombus by angiography or autopsy [21] |
| **Takotsubo cardiomyopathy**: Transient regional wall motion abnormalities of left (LV) or right ventricle myocardium that extend beyond a single epicardial vascular distribution and often result in circumferential dysfunction of the ventricular segments involved. Culprit atherosclerotic coronary artery disease or other pathological conditions to explain the pattern of temporary LV dysfunction are absent. There are new and reversible ECG abnormalities and significantly elevated serum natriuretic peptide during the acute phase [22]. |
| **Arrhythmias with new onset during the NCCU-stay**, i.e. atrial fibrillation, supraventricular tachycardia, ventricular tachycardia, atrio-ventricular blockage. |
| **Survived cardiac arrest** following cardiopulmonary resuscitation after a sudden loss of cardiac function. |

**Figure 1**: **Timing of onset of cardiac complications.**
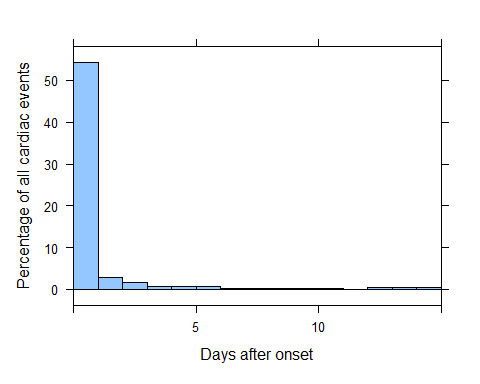


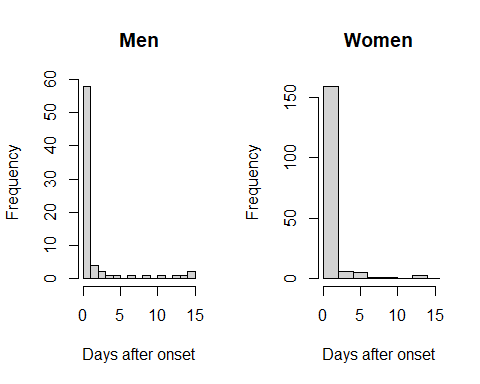


**Figure 1**: Histograms showing the relative amount of all patients with cardiac complications per day after aneurysmal subarachnoid hemorrhage. The panel above refers to the all population. The panel below shows the timing of onset of cardiac complications by sex.

**Table 2.** **Demographics, pre-existing cardiac conditions, cardiovascular risk factors stratified by patients with vs. without cardiac complications.**

|  | Overall | No | Yes | p value |
| --- | --- | --- | --- | --- |
| Patients with cardiac complications (n) | 387 | 137 | 250 |  |
| Sex |  |  |  | **<0.001** |
| Female n (%) | 251 (64.9) | 74 (54.0) | 177 (70.8) |  |
| Male n (%) | 136 (35.1) | 63 (46.0) | 73 (29.2) |  |
| Age, years (median [Q1-Q3]) | 57.00 [49.00 - 68.00] | 52.00 [45.00 - 59.00] | 61.00 [52.00 - 70.75] | **<0.001** |
| NCCU-LOS days (median [Q1-Q3]) | 15.00 [11.00 - 22.00] | 13.00 [11.00 - 16.00] | 17.00 [12.00 - 27.00] | <0.001 |
| CCI total (median [Q1-Q3]) | 0.00 [0.00 - 2.00] | 0.00 [0.00 - 1.00] | 1.00 [0.00 - 2.00] | **<0.001** |
| History of any heart disease (%) | 184 (47.8) | 42 (31.1) | 142 (56.8) | **<0.001** |
| Coronary heart disease (%) | 12 (3.1) | 2 (1.5) | 10 (4.0) | 0.294 |
| Heart failure (%) | 1 (0.3) | 0 (0.0) | 1 (0.4) | 1.000 |
| Arterial hypertension (%) | 174 (45.2) | 39 (28.9) | 135 (54.0) | **<0.001** |
| Valvular disease (%) | 16 (4.2) | 1 (0.7) | 15 (6.0) | **0.028** |
| Rhythmogenic heart disease (%) | 10 (2.6) | 2 (1.5) | 8 (3.2) | 0.495 |
| Current smoking (%) | 178 (53.8) | 67 (54.0) | 111 (53.6) | 1.000 |
| Alcohol use disorder (%) | 13 (3.4) | 5 (3.6) | 8 (3.2) | 1.000 |

**Table 2:** Demographics, pre-existing cardiac conditions, cardiovascular risk factors stratified by patients with vs. without cardiac abnormalities. Comparisons among patients whit and without cardiac complications. Abbreviations: NCCU-LOS length of stay at the neurocritical care unit; CCI Charlson Comorbidity Index, Q1-Q3 quartiles 1 and 3.

**Table 3: Aneurysm localization, severity scores, and intracranial complications stratified by cardiac complications.**

|  | Overall (N=387) | No (n=137) | Yes (n=250) | p value |
| --- | --- | --- | --- | --- |
| *Aneurysm localization* | |  |  | 0.992 |
| Internal carotid artery | 31 (8.0) | 13 (9.5) | 18 (7.2) |  |
| Middle cerebral artery | 96 (24.8) | 35 (25.5) | 61 (24.4) |  |
| Anterior communicating artery | 116 (30.0) | 41 (29.9) | 75 (30.0) |  |
| Anterior cerebral artery | 6 (1.6) | 3 (2.2) | 3 (1.2) |  |
| Vertebral artery | 12 (3.1) | 3 (2.2) | 9 (3.6) |  |
| Posterior cerebral artery | 4 (1.0) | 1 (0.7) | 3 (1.2) |  |
| Posterior inferior cerebellar artery | 20 (5.2) | 6 (4.4) | 14 (5.6) |  |
| Anterior inferior cerebellar artery | 1 (0.3) | 0 (0.0) | 1 (0.4) |  |
| Posterior communicating artery | 56 (14.5) | 20 (14.6) | 36 (14.4) |  |
| Pericallosal artery | 12 (3.1) | 5 (3.6) | 7 (2.8) |  |
| Basilar artery | 24 (6.2) | 7 (5.1) | 17 (6.8) |  |
| Superior cerebellar artery | 5 (1.3) | 2 (1.5) | 3 (1.2) |  |
| Anterior choroidal artery | 3 (0.8) | 1 (0.7) | 2 (0.8) |  |
| Unknown | 1 (0.3) | 0 (0.0) | 1 (0.4) |  |
| Anterior circulation (%) | 304 (78.6) | 116 (84.7) | 188 (75.2) | 0.041 |
| Posterior circulation (%) | 83 (21.4) | 33 (15.3) | 50 24.8) |  |
| *Bleeding severity scores* |  |  |  |  |
| WFNS (%) |  |  |  | **<0.001** |
| Score 1 | 126 (32.6) | 71 (51.8) | 55 (22.0) |  |
| Score 2 | 80 (20.7) | 28 (20.4) | 52 (20.8) |  |
| Score 3 | 21 (5.4) | 7 (5.1) | 14 (5.6) |  |
| Score 4 | 75 (19.4) | 13 (9.5) | 62 (24.8) |  |
| Score 5 | 84 (21.7) | 17 (12.4) | 67 (26.8) |  |
| NaN | 1 (0.3) | 1 (0.7) | 0 (0.0) |  |
| WFNS dichotomized (%) |  |  |  | **<0.001** |
| Low grade WFNS | 227 (58.8) | 106 (77.9) | 121 (48.4) |  |
| High grade WFNS | 160 (41.2) | 31 (22.1) | 129 (51.6)) |  |
| Hunt and Hess (%) |  |  |  | **<0.001** |
| 1 | 83 (21.5) | 46 (33.6) | 37 (14.9) |  |
| 2 | 96 (24.9) | 47 (34.3) | 49 (19.7) |  |
| 3 | 68 (17.6) | 18 (13.1) | 50 (20.1) |  |
| 4 | 67 (17.4) | 14 (10.2) | 53 (21.3) |  |
| 5 | 71 (18.4) | 11 ( 8.0) | 60 (24.1) |  |
| N/A | 1 ( 0.3) | 1 ( 0.7) | 0 ( 0.0) |  |
| mFisher (%) |  |  |  | **<0.001** |
| 1 | 19 ( 4.9) | 13 ( 9.5) | 6 ( 2.4) |  |
| 2 | 22 ( 5.7) | 12 ( 8.8) | 10 ( 4.0) |  |
| 3 | 97 (25.1) | 46 (33.6) | 51 (20.4) |  |
| 4 | 247 (63.8) | 65 (47.4) | 182 (72.8) |  |
| N/A | 2 ( 0.5) | 1 ( 0.7) | 1 ( 0.4) |  |
| *Radiological findings* |  |  |  |  |
| Intracerebral hemorrhage (%) | 104 (26.9) | 28 (20.4) | 76 (30.4) | **0.046** |
| Hydrocephalus/Ventriculomegaly (%) | 210 (54.3) | 50 (36.5) | 160 (64.0) | **<0.001** |
| Intraventricular hemorrhage (%) | 271 (70.2) | 78 (56.9) | 193 (77.2) | **<0.001** |
| Subdural hematoma (%) | 34 (8.8) | 11 (8.0) | 23 (9.2) | 0.840 |
| *Treatment modalities* | |  |  | 0.180 |
| EVD placement n (%) | 210 (54.3) | 49 (35.8) | 161 (64.4) | **<0.001** |
| Conservative | 14 (3.6) | 4 (2.9) | 10 (4.0) |  |
| Endovascular | 194 (50.1) | 61 (44.5) | 133 (53.2) |  |
| Surgical | 179 (46.3) | 72 (52.6) | 107 (42.8) |  |
| *Complications:* |  |  |  |  |
| Re-rupture (%) | 2 (0.5) | 0 (0.0) | 2 (0.8) | 0.758 |
| Radiological vasospasm (%) | 207 (60.0) | 74 (59.2) | 133 (60.5) | 0.909 |
| Vasospasm with perfusion deficits (%) | 105 (27.1) | 28 (20.4) | 77 (30.8) | **0.038** |
| Delayed cerebral ischemia (%) | 98 (28.4) | 28 (21.9) | 70 (32.3) | 0.052 |

**Table 3:** Aneurysm localization, severity scores, and intracranial complications. Comparisons among patients with and without cardiac complications. Abbreviations: WFNS World Federation of Neurosurgical Societies; EVD external ventricular drain.

**Table 4: Outcomes of patients with vs. without cardiac complications.**

|  | Overall (N=387) | No (n=137) | Yes (n=250) | p value |
| --- | --- | --- | --- | --- |
| Died in NCCU n (%) | 49 (12.7) | 10 (7.3) | 39 (15.6) | **0.029** |
| Died during follow-up n (%) | 106 (27.4) | 22 (16.1) | 84 (33.6) | **<0.001** |
| GOSE 1-3 months after onset (median [Q1-Q3]) | 5.00 [3.00 - 6.00] | 6.00 [4.00 - 7.00] | 4.00 [2.00 - 6.00] | **<0.001** |
| GOSE 1-3 months stratified |  |  |  | **<0.001** |
| Favorable Outcome n (%) | 201 (51.9) | 97 (70.8) | 104 (41.6) |  |
| Unfavorable Outcome n (%) | 186 (48.1) | 40 (29.2) | 14658.4) |  |
| GOSE 12 months after onset (median [Q1-Q3]) | 5.00 [3.00 - 7.00] | 7.00 [5.00 - 8.00] | 5.00 [1.00 - 7.00] | <0.001 |
| GOSE 12 months stratified |  |  |  | **<0.001** |
| Favorable Outcome n (%) | 211 (60.6) | 97 (76.4) | 114 (51.6) |  |
| Unfavorable Outcome n (%) | 176 (39.5) | 40 (23.6) | 136 (48.4) |  |

**Table 4:** Outcomes. Comparisons among patients with and without cardiac complications. Abbreviations: NCCU Neurocritical Care unit; GOSE Glasgow Outcome Scale.

**Figure 2. ROC Curves.**

**
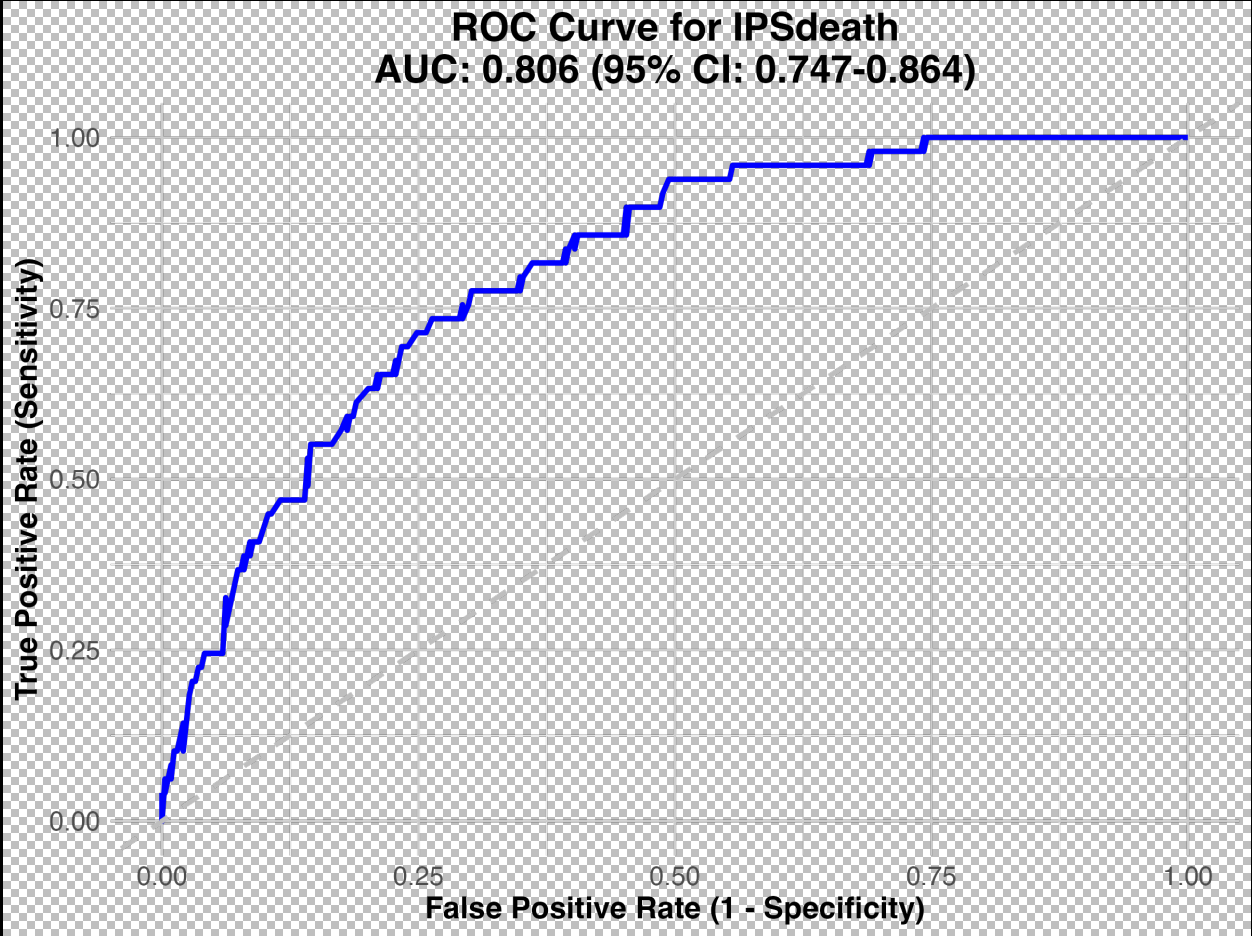
**

**
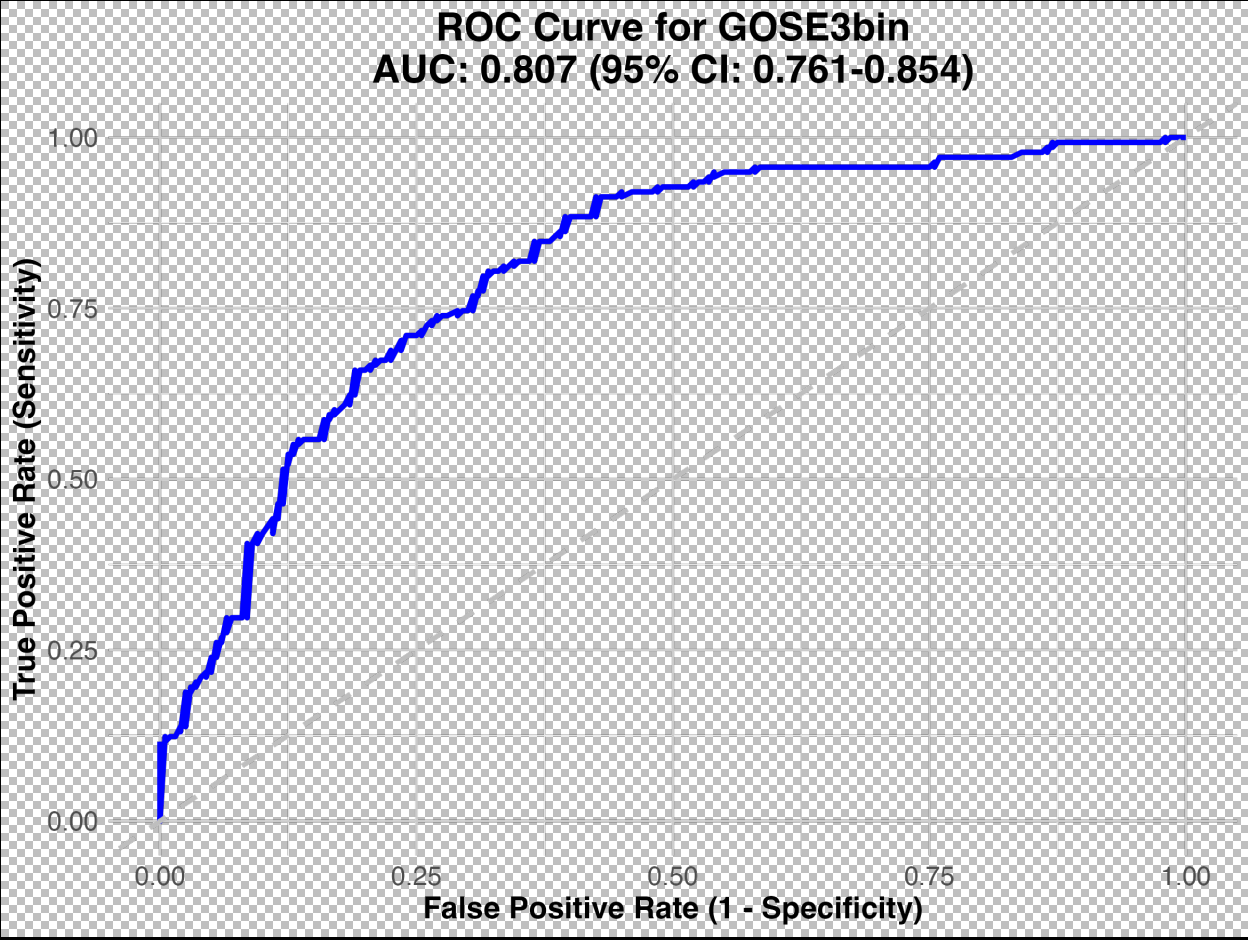
**

**
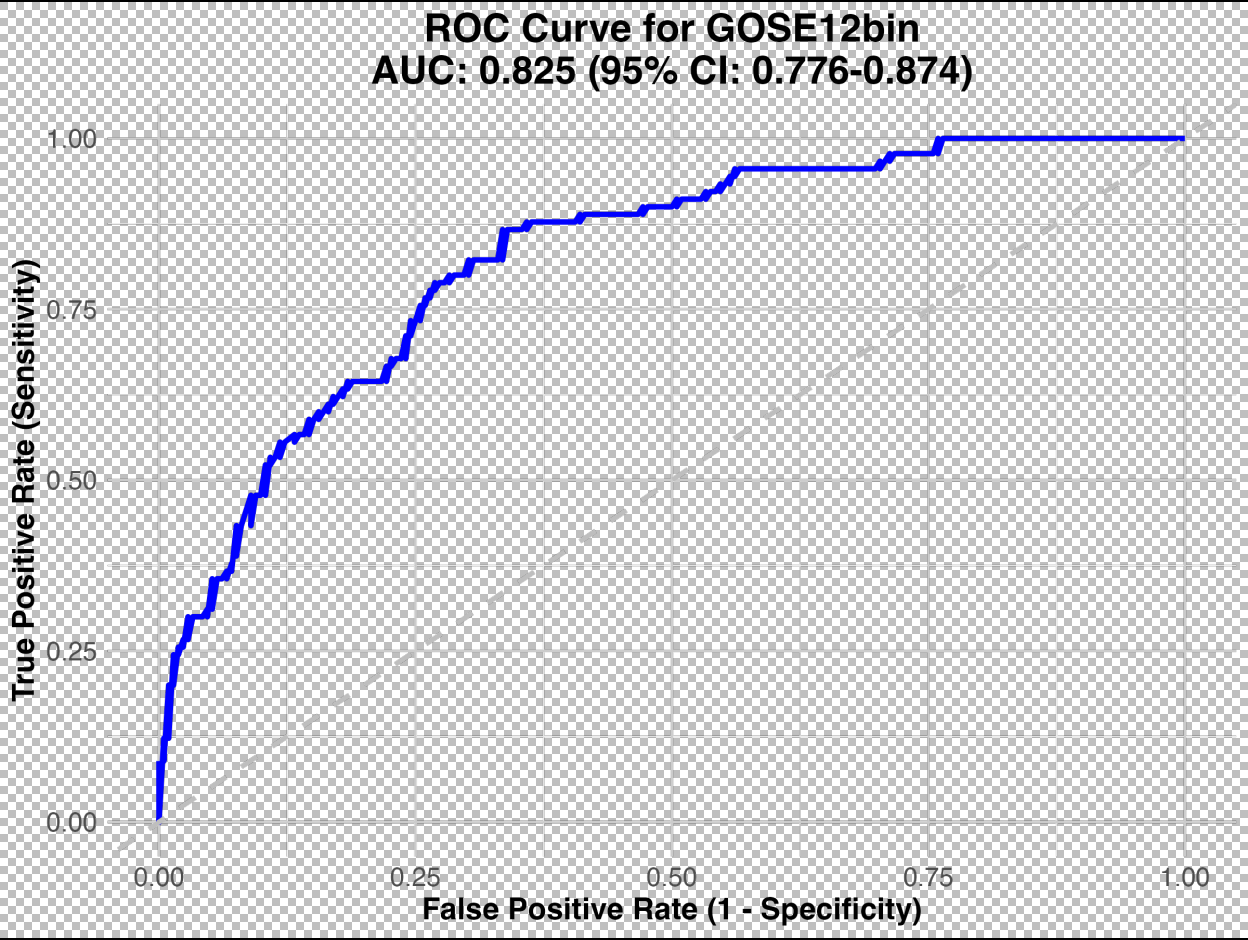
**

**Figure 2.** ROC analysis for NCCU-mortality (above), unfavorable outcomes at 3 months (in the middle), unfavorable outcomes at 12 months (below).
